# Predictors of Complications and Unfavorable Outcomes of Minimally Invasive Surgery Treatment in Elderly Patients With Degenerative Lumbar Spine Pathologies (Case Series)

**DOI:** 10.3389/fsurg.2022.869345

**Published:** 2022-04-26

**Authors:** Vladimir Klimov, Aleksey Evsyukov, Evgeniya Amelina, Sergey Ryabykh, Alexander Simonovich

**Affiliations:** ^1^European Medical Center, Moscow, Russia; ^2^Department of Neurosurgery, Peoples' Friendship University of Russia (RUDN University), Moscow, Russia; ^3^Division of Spinal Pathology and Rare Diseases, Ilizarov National Medical Research Centre for Traumatology and Orthopedics, Kurgan, Russia; ^4^Stream Data Analytics and Machine Learning Laboratory, Novosibirsk State University, Novosibirsk, Russia; ^5^Priorov National Medical Research Center of Traumatology and Orthopedics, Moscow, Russia; ^6^Research Organization Department, Y.L. Tsivyan Novosibirsk Research Institute of Traumatology and Orthopedics, Novosibirsk, Russia

**Keywords:** lumbar spine, degenerative disorders, minimally invasive surgery, complications, elderly patients, case series

## Abstract

**Introduction:**

The use of minimally invasive surgery (MIS) results in fewer adverse and more improved outcomes. However, the literature data describing the factors increasing the number of complications, reoperation frequency and unscheduled re-hospitalizations in older patients after MIS are contradictory. In this study, a large number of patients was investigated for the complications of minimally invasive surgical treatment of degenerative disease of the lumbar spine in older patients. The objective of the study was to determine the predictors of unfavorable outcomes in such patients.

**Materials and Methods:**

1,013 patients underwent MIS (decompression alone, TLIF, LLIF, ALIF) in 2013-2017. All operations were performed with the participation of the authors (neurosurgeons). The patient's average age was 66. The following data were collected: BMI; CCI; presence of postoperative complications according to the Dindo-Clavien classification; unplanned readmission at 90 days; hospital length of stay (LOS); surgical complexity (low, intermediate, and high); surgical time; and risk factors. The cumulative reoperation rate was determined at 5-years follow-up.

**Results:**

A total of 256 patients suffered a complication (25.2%), 226 classified as mild (grade I, II, IIIA), and 30 - as severe (IIIB, IVA). Such factors as the surgical complexity, BMI > 30, surgical time, number of operated levels were associated with a significant risk of developing a complication. For patients with and without complications, LOS was 9.3 and 6.3 days, respectively (*p* < 0.0001), the unplanned readmission rate was 1.3%. 104 patients underwent 133 revision operations. The 5-year cumulative reoperation rate was 15.2%, and the reoperation index was 12.1%. The CCI had no statistically significant effect on the complication incidence after MIS. A higher risk of complications was found in patients who underwent intermediate-complexity surgery (MIS TLIF) compared with uncompounded (decompression alone) and more complex (MIS LLIF, MIS ALIF) surgical procedures (*p* < 0.001 and *p* = 0.001, respectively).

**Conclusion:**

A register of postoperative complications is an important tool for health quality assessment and choosing the best surgical option that helps to establish measures to reduce such complications. Using MIS for the treatment of elderly patients reduces the number of severe complications.

## Introduction

The problem of improving quality of life for elderly and geriatric patients with degenerative lumbar spine pathologies of is a matter of ongoing scientific research that still remains unresolved. The relevance of the issue is explained by increasing life expectancy, advancements in diagnostics of this complex disease, availability of new data on its etiology and pathogenesis, and scientific and technical progress leading to the development of new surgical options (1). The complexity of the issue rises from the nosological diversity of degenerative pathology; the possibility of multiple and multilevel degenerative changes in the spine; the non-homogeneity of the elderly patient population, and the somatic comorbidities (2). Most patients have excess body mass, reduced bone tissue mineral density, comorbidity burden, and various degrees of sagittal imbalance of the vertebral column with respective compensatory mechanisms. All these factors make it difficult to come up with unified assessment criteria and identify an optimal surgical treatment. Surgical interventions come with risks of intraoperative and postoperative complications, the complication rate naturally increasing with age and the presence of a concomitant pathology. However, the literature data on complication predictors are contradictory (3–8). Several papers dedicated to identifying predictors of unfavorable surgical outcomes in elderly patients with degenerative LSS are available, but the conclusions regarding the effect of obesity, concomitant pathology, and psychological status of patients turn out to be ambiguous (9–13). Evidence-based studies are available showing an increase in complication rate in elderly and geriatric patients receiving surgical treatment of longer duration (14). Some authors call for using maximum clinical efficacy to identify the indications, restrictions, and extent of surgical treatment (4). Others note the high success rate of surgical treatment after using fixation hardware in elderly patients (15).

Ambiguous long-term results of surgical treatment in this age group call for further study and multi-aspect assessment. Thus, the objective of the present paper was to study predictors of complications and unfavorable outcomes after minimally invasive surgical (MIS) treatment of degenerative lumbar spine pathology in elderly patients.

## Materials and Methods

Retrospective analysis of the effect of concomitant medical conditions, including obesity and osteoporosis, in a consecutive case series of 1,013 elderly and geriatric patients (WHO classification of 1963) operated for degenerative lumbar spine pathology was performed at single-center Federal Neurosurgical Center (Novosibirsk, Russia). The data were collected from December 1, 2013 to December 31, 2017. The analysis included 367 (36%) male and 646 (64%) female patients, the average age being 66 years [66/65 (62;69) years]. Here and below, the data format is as follows: mean/median (quartile 1; 3).

### Inclusion Criteria

1. Elderly and geriatric age under the WHO classification of 1963;
2. Degenerative changes in the lumbar spine, the cause(s) being as follows:
  - Disc herniation and/or spinal canal stenosis;
  - Segmental instability and/or spinal deformity in sagittal (low grade (Meyerding grade 1–2) degenerative spondylolisthesis) (16) and frontal (Cobb angles of >10° and ≤30°) planes;
  - Respective clinical manifestations in the form of intermittent neurogenic claudication, radiculopathy or both combined or persistent spinal pain syndrome in the form of chronic pains in the lumbar spine (VAS score of 5 or above);
3. No positive effect from combined conservative therapy for 12 weeks;
4. Minimally invasive surgical treatment of degenerative disease (MIS).

The patients with idiopathic scoliosis, degenerative scoliosis in the lumbar spine with a Cobb angle of over 30°, and the patients with previous history of spinal surgery were not included in the analysis. Exclusion criteria also included spinal tumors and inflammatory lesions, decompensated somatic pathology, and intermittent claudication of vascular origin.

The 1,013 patients were operated minimally invasive techniques (decompression alone, TLIF, LLIF, ALIF) in 2013–2017.

### Assessment Methods and Criteria

Taking into account the age of the patients, a complete standard examination was performed in the outpatient setting to discover somatic comorbidities, and the patients received the respective treatment, if necessary. A preoperative neurological clinical examination consisting of X-ray imaging of all spinal regions in standard views with frontal and sagittal balance assessment, an intrathecal contrast-enhanced CT scan of the lumbar spine, and an MRI scan was performed. At *T*-values below −2.5A, a mandatory DXA scan of the lumbar spine and proximal thigh was performed, and bone cement injection was added to screw fixation.

Lumbar spine radiography with functional tests (maximum flexion and extension) was performed to describe segmental biomechanics as follows: vertebral displacement in the neutral position, sagittal translation and angulation. Vertebral displacement was measured according to White and Panjabi (17).

A lumbar spine MRI scan was performed for all patients to evaluate degenerative changes in the intervertebral discs. Post-operative MRI scans were performed, when patients showed signs of complications as follows: CSF leak, epidural hematoma, residual nerve root compression, early and late disc reherniation, and surgical site infection.

A spiral CT scan (SCT) with intrathecal contrast enhancement (omnipaque 300, 10 ml) and with further MPR and 3D VRT reconstruction was also performed for all patients to specify the stenotic region. Screw malposition was evaluated according to Rao et al. (18).

The Charlson Comorbidity Index (CCI) (19) reflecting a 10-year survival rate in patients with concomitant somatic pathology adjusted for age was calculated to evaluate the somatic status and concomitant somatic pathology (20).

The effect obesity and concomitant somatic pathologies had on the quality of life, functional status, and pain syndrome was analyzed taking into account surgical time (min), intraoperative blood loss (ml), hospital length of stay (bed-day), early and late postoperative complications (21).

The VAS score was used to evaluate pain intensity (in legs and back) before and after the operation and also at 12 and 24 months after each operation. The Oswestry Disability Index (ODI) and SF36 form were used to evaluate the functional activity and quality of life in patients (PH is physical health and MH is mental health) before the operation, at 12 and 24 months after the operation, and during future visits. Changes were evaluated based on MCID (22).

The clinical effectiveness of the surgical treatment was evaluated in the form of an in-person or a phone survey. The patients were invited for in-person orthopedic and neurological examination or inpatient examination, if necessary. 748 (74%) patients underwent follow-up examination. The other patients either did not answer the phone call or refused to come to examination due to living too faraway.

Complications were evaluated based on the Dindo-Clavien classification (2004) (23) validated for lumbar spine surgery (24).

### Assessment of Long-Term Results

Vertical X-rays and CT scans from the early postoperative period were used to specify the positions of hardware elements. Plain and functional X-rays and CT scans performed at 3, 6, and 12 months after the operation were used to control the hardware positions and identify instability in the operated and adjacent segments. Bone block formation according to Tan (presence at Grades 1–2 and absence at Grades 3–4) (25) was evaluated based on CT scans at 12 months after the operation. After that, X-ray imaging and CT scans were performed, if necessary.

Cumulative reoperation rate. This parameter was calculated for a 5-year period as the sum of yearly reoperation rates in patients with sufficient follow-up period (here, *n* = 5):


$$\begin{array}{l} I_{n}=\overset{n}{\underset{i=1}{\sum }}\frac{Number\;of\;reoperations\;at\;the\;i-th\;year\;after\;a\;primary\;procedure\;}{Number\;of\;patients\;followed-up\;for\;at\;least\;i\;years},\%. \end{array}$$

The reoperation frequency index for *n* years was calculated similarly as the sum of yearly numbers of reoperated patients for patients with sufficient follow-up period (26).


$$\begin{array}{l} P_{n}=\overset{n}{\underset{i=1}{\sum }}\frac{Number\;of\;the\;first\;reoperations\;at\;the\;i-th\;year\;}{Number\;of\;patients\;followed-up\;for\;at\;least\;i\;years},\%. \end{array}$$

Generally, the reoperation frequency ≤ the cumulative reoperation rate.

### Clinical and Radiographic Description of the Patient Cohort

The main reason for the patients to seek for medical help was neurological compression syndromes and persistent spinal pain syndrome. The leg-pain VAS scores were 6.7/7 (5; 8). The spine pains with VAS scores of 6.1/6 (5; 8) had a significant functional and quality-of-life impact with a mean ODI score of 56.2/57 (44; 66), SF-36 PH score of 26.6/26 (23; 30), and SF-36 MH score of 27.9/27 (22; 33). Leg pains prevailed over back pains in 406 (40%) patients. Back pains prevailed in 262 (26 %) patients, of which 29 identified a spinal pain syndrome with leg-pain VAS score not exceeding 2 as the only quality of life impact. The same pain intensity in the legs and back was observed in 345 (34%) patients.

Neurological clinical examination showed radiculopathy in 665 (68%) patients with leg- pain VAS score of 7/7 (6; 8). Intermittent neurogenic claudication was observed in 319 (31%) patients with walking distances of 96/100 (50; 100) m. Most patients (883 (87%) had single-level lumbar spine stenosis; clinically significant spinal canal stenosis at two levels was identified in 108 (11%) patients and at three levels in 22 (2%) patients.

Neuroimaging showed degenerative spondylolisthesis in 428 (42%) patients, of which 390 (91%) were grade 1 cases, and 38 (9%)—grade 2 cases, according to Meyerding (16). In addition, there were 81 (12.8%) cases, where degenerative spondylolisthesis was not accompanied by segmental instability, i.e., the score was below 5, according to A. A. White and M. M. Panjabi (17). The scores of 5 and above corresponding to clinical segmental instability were observed in 338 patients (33% of the total cohort size). The instability was accompanied by radiculopathy, intermittent neurogenic claudication or both in 317 patients (93.8% of 338). It involved a single segment in 307 (90.8%) cases, two segments in 30 (8.9%) cases, and three segments in 1 (0.3%) case. A pathology in the apical segment of lumbar lordosis at L4-L5 observed in 243 patients (71.9%) was the most common with a mean score of 6.3/6 (6; 7), according to A. A. White and M. M. Panjabi.

Clinically significant spinal canal stenosis without segmental instability was considered an indication for neurovascular structures decompression (27).

A total of 365 microsurgical discectomies were performed, of which 356 were at a single level, 8—at two levels, and 1—at three levels. Microsurgical decompression using the modified Wiltse approach was performed in 28 cases. A total of 86 unilateral microsurgical decompressions of lateral recess were performed in patients with lateral stenosis, of which 83 were at a single level and 3—at two levels. Lateral recess decompression was combined with microsurgical discectomy in 23 patients. Over-the-top microsurgical decompression was performed in 145 patients with clinical and radiographic signs of central canal stenosis.

Stabilization surgery (28) was performed in patients with segmental instability according to White and Panjabi (17). MIS TLIF with direct over-the-top decompression and transpedicular fixation (TPF) was performed in 142 out of 163 central canal stenosis cases, and indirect decompression (ALIF/LLIF)—in 21 cases. MIS TLIF with direct microsurgical decompression was performed in all patients with clinically significant lateral canal stenosis (*n* = 103) (29). MIS TLIF was performed in 40 out of 51 patients with foraminal stenosis, and ALIF/LLIF in 11 patients. MIS TLIF was used for surgical treatment of instability in 294 patients, ALIF—in 23 patients, and LLIF—in 21 patients. Operations were performed at a single level in 307 cases, on two levels—in 30 cases, and at three levels—in 1 case. All multilevel surgical procedures were MIS TLIF operations.

Degenerative scoliosis of the lumbar spine with a Cobb angle of 10–30° in the frontal plane was diagnosed in 91 (9%) patients. Only 51 patients from the studied cohort or 5% of the total cohort size received two-stage corrective operations with two MIS techniques (LLIF and MIS TPF) performed in one surgical session. Among those, 44 patients (86%) were female, and 7 patients (14%) were male. The mean age was 67/67 (63; 70) years (from 60 to 81 years). All these patients had a disability with an ODI score of 56/54 (45; 62) accompanied by quality-of-life deterioration on the SF-36 scale [PH score of 25/24 (22; 28), MH score of 26/26 (22; 28)]. The Cobb angle for the group was 16.5/15 (11; 20°). Patients suffered from spinal pain syndrome with a spine-pain VAS score of 6.6/6 (5; 8) and various compression syndromes [84% or 43 patients in the third group had a leg-pain VAS score of 6.3/6 (5; 8)]. All patients complained about the inability to stand upright for a long time and walking impairments due to difficulty in maintaining vertical position as a result of local or global sagittal balance impairment. Pain syndrome was caused by degenerative scoliosis in the lumbar spine [mean Cobb angle of 16.5/15 (11; 20°)]. All patients from this group had an N curve as per the SRS-Schwab classification. All patients had sagittal balance impairments with excessive sagittal modifier values. Neuroimaging showed that 35 patients from the third group (68.6%) had degenerative scoliosis combined with degenerative spondylolisthesis at one (9), two (23), or three levels (3). Among those, 34 were a Meyerding grade 1 case, and one—a grade 2 case. The remaining 40 patients (4%) received decompressive surgery or decompression with spine stabilization depending on the prevalent clinical symptoms.

Most patients had excess body mass (30), with 327 (32%) patients having BMI above the normal threshold of 25 but below 30 (25 ≤ BMI < 30). Class 1 obesity (30 ≤ BMI < 35) was observed in 322 (32%) patients, class 2 obesity (35 ≤ BMI < 40)—in 181 (18%) patients, and class 3 obesity (BMI > 40)—in 79 (8%) patients.

Somatic comorbidity was discovered in 999 (98.6%) patients: 155 patients (15.5% from 999) had an isolated pathology, while the vast majority of 843 patients (84.5% from 999) had a concomitant pathology. Gastrointestinal and cardiovascular pathologies were the most common. The mean CCI value in the studied cohort was 63/77 (53; 90%).

### Statistical Analysis

The normal distribution hypothesis for quantitative values was tested using the Kolmogorov–Smirnov and Shapiro–Wilk tests. Since most quantitative and scale data were not normally distributed, nonparametric criteria were used in the calculations. In the present paper, the numerical data format is as follows: mean/median (quartile 25; 75%).

The correlations between values and their strengths were estimated using Spearman's correlation coefficient. The two-sided Mann–Whitney test was used to compare two independent samples by quantitative values, and the Kruskal–Wallis test—for three samples. To compare the studied groups by qualitative values, Fisher's exact test or its asymptotic version (for contingency table dimensions above 2 × 2) was applied.

To isolate complication predictors, a logistic regression model with a stepwise algorithm for predictor inclusion/exclusion was used. The prediction adequacy hypothesis was tested using the Hosmer–Lemeshow test. R software was used for statistical data processing (31).

This case series has been reported in line with the PROCESS Guideline (32).

## Results

The surgical procedures (*n* = 1,146) were divided into three types 3 based on surgical injury and extent of operation:

Patients from the first group (*n* = 624) underwent low-complexity operations, such as microsurgical discectomy, lateral recess decompression, central canal decompression, including both single-level and multilevel operations. The low-complexity group included 624 patients (61.6% of the total cohort size): 277 males (44.4%) and 347 females (55.6%) aged 60–88 years [67/65 (62; 70)]. A total of 710 operations were performed, including 86 reoperations.

Patients from the second group (*n* = 338) underwent intermediate-complexity operations, such as direct decompression with spinal fusion and fixation (mostly at 1 and 2 levels with one operation at three levels). This group included 338 patients (33% of the total cohort size): 83 males (24.6%) and 255 females (75.4%) aged 60–86 years [66/65 (62; 68)]. The patients from the second group underwent a total of 379 operations, including 41 reoperations.

Patients from the third group (*n* = 51) underwent high-complexity operations using two surgical approaches, such as ALIF and LLIF with TPF, in one session. The total number of operations in this group included 51 initial operations and 6 reoperations.

### Clinical Effectiveness

The mean hospital length of stay after MIS decompression alone in patients from the first group was 5.6 bed-days, intraoperative blood loss−117/50 (50; 100) ml, surgical time−84/75 (60; 100) min. Table 1 shows the treatment results in 262 patients assessed at long-term follow-up. The complete assessment of all clinical and radiographic criteria after treatment was performed in 423 (68%) patients from the first group. Some patients were included in the assessment several times during the present study. The cumulative 5-year reoperation rate in the first group was 15.3%, and reoperation frequency−12.6%.

**Table 1:** Quality-of-life assessment in patients from the first group (*marks statistically significant changes (*p* < 0.05) compared to preoperative values).

**Parameters**	**Pre-op (624)**	**Up to 1 year (245)**	**Over 1 year (262)**
**Group 1** ODI	55/56 (42; 68)	27/27 (14; 38)*	18/13 (4; 29)*
Patients with ↓ ODI of at least 12.8 (22)	—	166	206
SF-36 PH	27/26 (23; 30)	40/39 (32; 47)*	43/46 (36; 52)*
Patients with ↑ SF-36 PH of at least 4.9 (22)	—	178	203
SF-36 MH	29/28 (22; 34)	41/42 (33; 51)*	46/50 (39; 54)*
Patients with ↑ SF-36 MH of at least 4.9 (22)	—	157	188

In the second group, the mean LOS was 8.3 days due to MIS. Surgical time of MIS TLIF direct decompressions [189/180 (150; 215)] was significantly longer compared to indirect decompressions with spinal stabilization [156/155 (130; 176)], the overall mean value being 184 min. Intraoperative blood loss was higher in TLIF operations, the overall mean value in the second group being 277/200 (100; 300) ml due to the use of MIS techniques. Assessment of all clinical criteria and quality-of-life indicators was performed in 282 patients (83%) from the second group. The assessment period was 28.9/24 (15; 41.8) months, see Table 2. The cumulative 5-year reoperation rate in the second group was 14.3%, and reoperation frequency 10.2%.

**Table 2:** Quality-of-life indicators in patients from the second group (*marks statistically significant changes (*p* < 0.05) compared to pre-op values).

**Parameters**	**Pre-op (338)**	**Up to 1 year (213)**	**Over 1 year (111)**
**Group 2** ODI	58/60 (52; 66)	26/24 (16; 36)*	24/22 (6; 38)*
Patients with ↓ ODI of at least 12.8 (22)	—	181	80
SF-36 PH	26/26 (22; 30)	38/38 (32; 45)*	41/43 (32; 51)*
Patients with ↑ SF-36 PH of at least 4.9 (22)	—	156	76
SF-36 MH	27/26 (20; 32)	41/38 (33; 53)*	44/49 (35; 54)*
Patients with ↑ SF-36 MH of at least 4.9 (22)	—	144	70

The mean LOS in the third group was 8.6 days due to the use of MIS techniques in all patients. Intraoperative blood loss was 260/200 (150; 275) ml. Although the surgical time for two-stage operations was 264/245 (210; 295) min, which is considered a predictor of a higher rate of severe complications (14) (surgical time > 180 min in geriatric patients), the use of MIS techniques for deformity correction made it possible to minimize the complication rate and reduce it to 31.4%. Long-term follow-up for 43 (84%) patients showed satisfactory results in most cases, see Table 3.

**Table 3:** Quality-of-life indicators in the long-term follow-up for patients from the third group (*marks statistically significant changes (p < 0.05) compared to preoperative values).

**Parameter**	**Pre-op (*n =* 51)**	**Long term (*n =* 43)**
**Group 3** ODI	56/54 (45; 62)	35/42 (22; 51)*
Patients with ↓ ODI of at least 12.8 (22)	—	38
SF-36 PH	25/24 (22; 28)	33/25 (24; 38)*
Patients with ↑ SF-36 PH of at least 4.9 (22)	—	38
SF-36 MH	26/26 (22; 28)	38/35 (28; 46)*
Patients with ↑ SF-36 MH of at least 4.9 (22)	—	37

### Complication Analysis

The surgical time, unplanned readmission within 90 days after the operation, and LOS (bed-days) were taken into account separately for the patients with and without complications, and cumulative complication rates were calculated for various surgical options. All revision operations performed in the follow-up period in the patient who underwent initial operations at our medical center were taken into consideration to calculate the cumulative reoperation rate.

A total of 256 complications (25.3% from 1.013) were registered, out of which 254 (25.1% from 1.013) were recorded within 90 days after the operation, see Table 4.

**Table 4:** Complications found following the Dindo-Clavien classification criteria (2004) in the three groups of patients.

**Type**	**Complication**	**Group 1**	**Group 2**	**Group 3**	**Total**
I	Blood loss of at least 500 ml	16	50	2	68
	Dural tear (no post-op CSF leak)	47	18	—	65
	Rao grade 1–2 lateral screw malposition	—	22	—	22
	Cortical endplate injury	—	8	1	9
	Cage migration	—	2	—	2
	Bone cement leakage to the spinal canal, paravertebral vein.	—	1	1	2
	Peritoneal injury	—	1	—	1
II	Increasing neurological deficit	6	8	3	17
	Blood loss of over 500 ml with blood transfusion		8	2	10
	Hematoma (epidural, retroperitoneal)	3	2	1	6
	Exacerbation of chronic urinary tract infection	2	2	1	5
	Superficial surgical site infection (SSI)	—	5	—	5
	Residual radicular pain syndrome	3	—	—	3
	Allergic response	1	—	—	1
	Acute psychoorganic syndrome	—	1	—	1
	Decompensated cardiovascular pathology	—	1	—	1
	Acute deep lower limb venous thrombosis	—	—	1	1
IIIA	Pharmacoresistant neuropathic pain syndrome	2	3	1	6
	Dural tear with CSF leak (external lumbar drainage)	—	1	—	1
IIIB	Short-term disc reherniation (up to 90 days)	8	—	—	8
	Epidural hematoma	4	—	—	4
	Incomplete decompression	3	1	—	4
	Rao grade 3 intracanal screw malposition (reoperation)	—	2	2	4
	Deep surgical site infection	1	2	—	3
	Short-term segmental instability (up to 90 days)	3	—	—	3
	Pseudoarthrosis (reoperation)	—	1	1	2
	Fixation hardware failure (reoperation)	—	1	—	1
IVA	Acute myocardial infarction	—	1	—	1
Total		99	141	16	256
		15.9%	41.7%	31.4%	25.3%

It is worth noting a small number of complications (31.4%) after two-stage corrective lumbar spine operations for degenerative scoliosis deformities in elderly patients with concomitant obesity and comorbidity burden.

*Cumulative reoperation rate* for lumbar spine operations in the third group of patients could not be assessed in the five-year follow-up period due to insufficient observation time, but the cumulative reoperation rate for the three-year follow-up was 7.8%, and so was reoperation frequency.

The total number of complications in the studied patient cohort was 256 (25.3%). Complications were significantly more rare in patients from group 1 compared to groups 2 and 3 (*p* < 0.001 and *p* = 0.01). All complications were divided into general, instrumentation-related, and neurological.

#### General Complications

Blood loss of at least 500.0 ml was the most common intraoperative complication (78 cases) amounting to 30.5% of the total number. Blood volume deficit was compensated by blood products (blood transfusion) in 10 of these cases. It is worth mentioning that in the vast majority of cases (58) this complication occurred in patients from group 2, who underwent TLIF. General complications also included intraoperative dural tears in 66 cases (25.8% of all complications), out of which in one case external CSF leak confirmed by MRI control was observed in the early postoperative period. The liquor fistula was closed in a course of conservative treatment combined with external lumbar drainage. Incidental durotomy was significantly more often observed in patients from group 1 (47 patients out of 66). Intraoperative dural suturing with adhesive sealing and local tissue grafting was performed in all patients with dural tears.

Slow (over 10 days) postoperative wound healing (superficial SSI) in patients with diabetes mellitus was observed in five cases. All wounds were healed by primary intention. Other short-term postoperative complications in the form of residual compression with clinical manifestations (4 cases) and long-term ones in the form of deep SSI (three cases), where postoperative wound revision was required, were observed only in groups 1 and 2. Residual radicular pain syndrome associated with nerve root traction injury in 3 patients from group 1 was reversed by conservative treatment. Within 90 days of the follow-up, early disc reherniation requiring hospital readmission and revision operation was observed in 8 patients from group 1 who underwent microsurgical discectomy (6 patients) and over-the-top decompression (2 patients). Clinical instability syndrome confirmed by functional radiography developed in 3 cases after spinal canal decompression for central stenosis, as a result of excessive resection of osseous and ligamentous structures. These patients received stabilization TLIF surgery.

Control MRI scans showed asymptomatic epidural hematomas in five cases fully lysed in the course of conservative treatment. They also showed retroperitoneal hematoma requiring follow-up and conservative treatment along the surgical approach in an early postoperative period after LLIF in one patient. Epidural hematomas were accompanied by clinical signs of cauda equina root compression requiring revision operation in 4 patients from group 1. All those patients received indirect coagulants long before the operation due to a concomitant cardiovascular pathology. Decompensated cardiovascular pathology (arterial hypertension), acute myocardial infarction, and deep left lower limb venous thrombosis were recorded in one case each. Conservative treatment was performed successfully in all cases. Acute psychoorganic syndrome in the form of delirium and allergic response was recorded in one case each as well. Exacerbation of urinary tract infection in the early postoperative period was discovered in 5 patients who received antibacterial therapy based on urine culture results and antibiotic sensitivity. Intraoperative peritoneum injury during ALIF surgery was recorded in one case, but it did not affect the postoperative course. Overall, general complications were recorded in 191 cases amounting to 74.6% of the total number of complications.

#### Instrumentation-Related Complications

Intraoperative vertebral body endplate injury was discovered in 9 cases, of which 5 during TLIF and 4 during LLIF. In one case, this instrumentation-related complication after TLIF led to pseudoarthrosis with interbody cage migration, which required a revision operation 10 weeks after the initial operation. Bone block formation was observed in 8 other cases in the long-term follow-up.

According to the postoperative MSCT scan, Rao grade 3 intracanal transpedicular screw malposition (18) requiring reoperation was discovered in four patients, and Rao grade 1–2 lateral screw malposition with no clinical signs was observed in 22 patients after TLIF surgery. Bone cement leakage with no clinical signs was recorded in two cases, of which one was spinal canal leakage, and one was paravertebral vein leakage. According to control MSCT scans, interbody cage migration with no clinical symptoms not requiring revision operation was discovered in two patients three months after the initial operation. Control examination showed hardware failure associated with pseudoarthrosis and requiring revision operation in two patients with recurrent spinal pain syndrome 7 and 11 months after the initial operation. Overall, 42 instrumentation-related complications amounting to 16.4% of the total number of complications were recorded.

#### Neurological Complications

An increasing neurological deficit in lower limbs was observed in the early postoperative period in 17 patients, 8 of them from group 2. In 14 of those cases, neurological status deterioration was caused by intraoperative nerve root retraction injury, which required long-term conservative therapy. In 3 cases, the post-LLIF complication was caused by surgical nerve root injury on the approach side. In two of those cases, improvement was observed in course of conservative therapy and rehabilitation activities. One female patient exhibited neurological deficit as persistent right hip flexor weakness on the approach side. Pharmacoresistant neuropathic pain syndrome developed within a year after the initial operation in six patients. All those patients responded positively to test stimulation, and chronic epidural stimulation systems were implanted. Overall, 23 neurological complications amounting to 9.0% of all complications were recorded.

It is worth noting that most complications (141) were recorded in group 2 accounting for 55.1% of all complications, which may be explained by the extent of the operations and their surgical technicalities.

Among a total of 256 complications, 254 (99%) developed within 90 days, 226 (88.3%) were identified as mild (Dindo-Clavien types I, II, and IIIA), and 30 (11.7%)—as severe (types IIIB and IVA). In 29 of the latter cases, reoperation under general anesthesia was required. The characteristics of patients with complications are presented in Table 5.

**Table 5:** Main characteristics of groups of patients with and without complications.

**Parameter**	**Without complications**	**With complications**	***p***
Number of patients	804	209	-
Age	66.2/65 (62; 69)	66.3/65 (62; 69)	0.992
Gender, % female	62%	70%	0.096
BMI	31.3/30.9 (27.6; 34.7)	32.5/31.7 (27.9; 36.9)	0.013
CCI, %	62.8/77 (53; 90)	64.2/77 (53; 90)	0.643
LOS (total within 90 days)	6.3/6 (5; 7)	9.3/7 (6; 11)	<0.0001
Primary LOS	6.3/6 (5; 7)	8.2/7 (6; 10)	<0.0001
Surgical time (total within 90 days)	113.7/90 (65; 150)	194./182,5 (125; 235)	<0.0001
Surgical time (initial)	113.7/90 (65; 150)	176.8/170 (110; 221.2)	<0.0001

Statistically significant differences were observed between these two groups in BMI (*p* = 0.013), which indicates an adverse effect of obesity on the complication rate. Significant differences were also identified in surgical time (initial operations took longer in the patients who later exhibited complications, *p* < 0.0001), and LOS (the number of bed-days was higher in patients with complications, *p* < 0.0001).

The distribution of complications (*n* = 256) with respect to operation complexity was as follows: 99 (38.7%) after low-complexity operations (decompression alone), 136 (53.1%) after intermediate-complexity operations (MIS TLIF), and 21 (8.2%) after high-complexity operations (ALIF and LLIF). The number of patients without complications (*n* = 804) after low-complexity operations was 536 (85.9%), after intermediate-complexity operations-−190 (64.6 %), and after high-complexity operations−78 (82.1%). Thus, a higher complication rate was identified in patients, who underwent intermediate-complexity operations (MIS TLIF), as opposed to low- and high-complexity surgical procedures (*p* < 0.001 and *p* = 0.001, respectively), which is probably due to higher risks of dural tears and intraoperative bleeding in direct microsurgical decompressions compared to indirect techniques.

The LOS (*n* = 1 013) after initial operation was 6.7/6 (5; 8) bed-days (0 to 31 days). LOS in patients without complications was 6.3/6 (5; 7) (1–19 days), and for patients with complications-−9.3/7 (6; 11) bed-days (2–31 days), the obtained differences being statistically significant (*p* < 0.0001).

### Effect of Excess Body Mass on Complication and Reoperation Rates

The effect of obesity and concomitant somatic pathology on the quality of life, functional status and pain syndrome, surgical time, LOS, blood loss parameters, reoperation rate, complication rate, and nature of complications was investigated. The obtained results were analyzed in the three groups of patients.

The BMI value in group 1 was 30.6/30.1 (26.8; 33.6), in group 2 - 33.2/33 (28.8; 36.6), and in group 3−32.9/33.5 (29.2; 36.3). In group 1, it was significantly lower than that in groups 2 and 3 (*p* < 0.001 and *p* = 0.002, respectively).

Obesity (BMI ≥ 30) was diagnosed in 582 (57.5%) patients from the studied cohort. In group 1, BMI values of 30 and above were recorded in 50.6% (316) cases, whereas in group 2—in 68.0% (230) patients, and in group 3—in 36 patients (70.6%). Thus, excess body mass (BMI ≥ 30) appears to be a risk factor of segmental instability and degenerative lumbar spine deformity in elderly patients.

Correlation dependences between BMI and intraoperative blood loss, surgical time and LOS are presented in Table 6.

**Table 6:** BMI effect on LOS, blood loss, surgical time (rs is Spearman's correlation coefficient, and *p* is a statistical significance level).

**Parameters**	**Group 1**		**Group 2**		**Group 3**	
**Value**	**rs**	***p***	**rs**	***p***	**rs**	***p***
LOS	0.15	0.0001	0.04	0.48	0.25	0.07
Surgical time	0.26	<0.0001	0.15	0.007	−0.04	0.76
Blood loss	0.22	0.0001	0.16	0.004	0.18	0.21

The increase in BMI correlates significantly with higher surgical time in groups 1 and 2, but this correlation does not hold for group 3 where higher surgical time is primarily explained by surgical technicalities. Similarly, higher intraoperative blood loss in groups 1 and 2 correlates with higher BMI, but the correlation does not hold for group 3 patients for the same reasons. The increase in LOS is associated with higher BMI only in group 1, but the correlation does not hold for groups 2 and 3.

Reoperation frequency dependences on BMI in different patient groups were compared. The number of patients requiring lumbar spine reoperation throughout the follow-up period and the number of patients with sufficient prospective follow-up time are shown in Table 7.

**Table 7:** Periodical reoperation frequency estimates and their dependence on BMI.

**Prospective follow-up time**	**BMI below 30**		**BMI of 30 and above**		**Comparison (*p*)**
**Group 1 (*****n** **=*** **624)**					
0–1 year	20 from 308	6.5%	22 from 316	7.0%	0.87
1–2 years	5 from 308	1.6%	10 from 316	3.2%	0.3
2–3 years	1 from 242	0.4%	5 from 259	1.9%	0.22
3–4 years	0 from 186	0.0%	1 from 194	0.5%	1
4–5 years	1 from 93	1.1%	3 from 106	2.80%	0.62
Total	9.6% in 5 years		15.4% in 5 years		—
**Group 2 (*****n** **=*** **338)**					
0–1 year	4 from 108	3.7%	7 from 230	3.0%	0.75
1–2 years	1 from 108	0.9 %	5 from 230	2.2%	0.67
2–3 years	1 from 78	1.3%	3 from 179	1.7%	1
3–4 years	0 from 58	0.0%	5 from 127	3.9%	0.33
4–5 years	0 from 33	0.0%	1 from 72	1.4%	1
Total	5.9 % in 5 years		12.2% in 5 years		—
**Group 3 (*****n** **=*** **51)**					
0–1 year	0 from 15	0.0%	4 from 36	10.0%	0.31
1–2 years	0 from 15	0.0%	0 from 36	0.0%	1
2–3 years	0 from 8	0.0%	0 from 21	0.0%	1
3–4 years	0 from 4	0.0%	1 from 10	10.0%	—
4–5 years	0 from 0	—	1 from 1	—	—
Total	not enough data		not enough data		—

Analysis of the correlation between BMI and reoperation period in the studied groups showed that reoperations within 1 year after the initial operation were not associated with BMI. However, the reoperations performed 2 or 3 years after the initial operation were often in patients with increased BMI. The BMI effect of on reoperation frequency was analyzed in two periods: up to 12 months and after 12 months.

The patients were divided into two groups based on extent of operation and surgical injury and comparable BMI values as follows: local microsurgical decompressions (624 operations) and decompressive operations with spinal stabilization and corrective operations (389 operations). The data on BMI values for patients with and without reoperations are presented in Table 8.

**Table 8:** BMI comparison for patients with and without reoperations.

**Postoperative period**	**BMI (initial operation) for patients without reoperations in the period of interest (1)**	**BMI (initial operation) for patients with reoperations (2)**	**BMI (reoperation) for patients with reoperations (3)**	**Statistical significance p, comparison (1) – (3)**	**Statistical significance p, comparison (1) – (2)**
**Local microsurgical decompressions (*****n** **=*** **624)**					
Up to 1 year	30.5/30.0 (26.7; 33.3)	31.1/30.4 (27.2; 34.4)	30.2/30.1 (26.6; 34)	*p =* 0.86	*p =* 0.52
After 1 year		32.6/32.9 (28.5; 35.9)	32.7/32.5 (29.4; 36.8)	*p =* 0.02	*p =* 0.03
**Decompressions with spinal stabilization and corrective operations (*****n** **=*** **389)**					
Up to 1 year	33.0/32.8 (28.5; 36.4)	32.7/33.8 (30.1; 35)	32.2/31.5 (27.6; 36.6)	*p =* 0.72	*p =* 0.96
After 1 year		35.6/36.2 (32.6; 38.1)	34.6/35.3 (31.4; 38.1)	*p =* 0.13	*p =* 0.03

Regardless of operation extent and surgical injury, BMI showed no effect on reoperation frequency within the first 12 months after the initial operation. It is primarily explained by the fact that vast majority of reoperations (73.7%) within the first follow-up year were caused by postoperative complications (residual compression, epidural hematomas, early disc reherniation, transpedicular screw malposition, cage migration, etc.). However, from the second follow-up year onward lumbar spine reoperation frequency was significantly higher in patients with obesity both after local microsurgical decompressions and decompressions with spinal stabilizations and corrective operations, statistical significance level being the same (*p* = 0.03). It is worth noting that patients after operations with hardware placement tended to lose some weight after the initial operation (lower BMI before the reoperation, *p* = 0.058), whereas patients after decompressions tended to have almost the same mean BMI before reoperation, despite weight loss recommendations given before hospital discharge.

Analysis of BMI correlation with quality of life, functional adaptation, and postoperative back and lower limb pain intensity within the first year after the initial operation showed the lack of correlation between the parameters of interest, see Table 9.

**Table 9:** BMI effect on quality of life (rs is Spearman's correlation, *p* is statistical significance level).

**Parameters**	**Pre-op**	**0–1 year**	**1–2 years**	**2–3 years**	**3–4 years**
VAS spine	0.09 (*p =* 0.04)	0.03 (*p =* 0.53)	0.25 (*p* < 0,001)	0.14 (*p =* 0.24)	0.28 (*p =* 0.01)
VAS leg	−0.03 (*p =* 0.50)	0.01 (*p =* 0.82)	0.30 (*p* < 0.001)	0.14 (*p =* 0.24)	0.14 (*p =* 0.22)
ODI	0.02 (*p =* 0.63)	0.09 (*p =* 0.07)	0.27 (*p* < 0.001)	0.12 (*p =* 0.3)	0.26 (*p =* 0.02)
SF-36 PH	0 (*p* = 0.99)	−0.06 (*p =* 0.26)	−0.28 (*p =* 0.001)	−0.19 (*p =* 0.18)	−0.34 (*p =* 0.01)
SF-36 MH	0 (*p =* 0.95)	−0.08 (*p =* 0.14)	−0.22 (*p =* 0.01)	−0.15 (*p =* 0.3)	−0.27 (*p =* 0.06)

However, a significant adverse effect of increased BMI on lower limb and back pain indicators, functional adaptation, and quality of life is determined after 1 year from the initial operation was observed. Despite some degree of irregularity in the long term, the adverse effect of BMI on pain intensity and quality of life remained a clear trend.

Distribution of complications according to the Dindo-Clavien classification across groups of the patients with BMI values below and over 30 is presented in Table 10.

**Table 10:** Complication distribution as per the Dindo-Clavien classification across groups of the patients with and without obesity.

**Type**	**Complication**	**BMI < 30**	**BMI ≥ 30**	**Total**
I	Blood loss of at least 500 ml	22	46	68
	Dural tear (no post-op CSF leak)	31	34	65
	Rao grade 1–2 lateral screw malposition	5	17	22
	Cortical endplate injury	4	5	9
	Cage migration	—	2	2
	Bone cement leakage to spinal canal, paravertebral vein.	—	2	2
	Peritoneal injury		1	1
II	Increasing neurological deficit	7	10	17
	Blood loss of over 500 ml with blood transfusion	2	8	10
	Hematoma (epidural, retroperitoneal)	1	5	6
	Exacerbation of chronic urinary tract infection	1	4	5
	Superficial SSI	1	4	5
	Residual radicular pain syndrome due to incomplete decompression	1	2	3
	Allergic response		1	1
	Acute psychoorganic syndrome	1		1
	Decompensated cardiovascular pathology		1	1
	Acute deep lower limb venous thrombosis		1	1
IIIA	Pharmacoresistant neuropathic pain syndrome	2	4	6
	Dural tear with CSF leak (external lumbar drainage)	—	1	1
IIIB	Short-term disc reherniation (up to 90 days)	3	5	8
	Epidural hematoma	3	1	4
	Incomplete decompression	1	3	4
	Rao grade 3 intracanal screw malposition (reoperation)	—	4	4
	Deep SSI	1	2	3
IIIB	Short-term segmental instability (up to 90 days)	2	1	3
	Pseudoarthrosis (reoperation)	1	1	2
	Fixation hardware failure (reoperation)	—	1	1
IVA	Acute myocardial infarction	1	—	1
Total		90	166	256
		20.9%	28.5%	25.3%

Comparison of complication rates in the patients with various BMI values showed higher rates for patients with obesity (*p* = 0.007).

A multifactor logistic regression model was used to identify complication predictors among the factors as follows: age, gender, BMI, and operation complexity based on surgical time and surgical injury estimates. A final regression model included only two factors: BMI and operation type (decompression alone, TLIF, ALIF/LLIF).

Quality metrics of the regression model were as follows: AUC = 0.64, sensitivity = 0.60, specificity = 0.69. The obtained Odd Ratios described the risks as follows:

- When the patient's BMI increased by 1, the complication rate was multiplied by 1.003 if operation type was the same;
- Complication rate for TLIF compared to decompression was multiplied by 1.223 (22.3% higher) if BMI value was the same;
- Complication rate for ALIF, LLIF compared to decompression was multiplied by 1.031 (3.1% higher) if BMI value is the same.

The obtained data showed the complication rate for TLIF surgery in patients with obesity increased by 22.3% compared to decompression, whereas for indirect decompression techniques, such as ALIF and LLIF, the complication rate only increased by 3.1 %.

Thus, obesity in elderly patients increases the probability of clinical instability syndrome that manifests in most cases in the form of degenerative spondylolisthesis with clinically significant spinal canal stenosis. In these cases, direct nerve root decompression, spondylolisthesis reduction, and rigid transpedicular fixation are required to eventually increase the complication rates, even when MIS techniques are used.

### Comorbidity Index Effect of on Complication and Reoperation Rates

The effect of the Charlson Comorbidity Index (CCI) on surgical treatment results in elderly patients with degenerative lumbar spine pathology was investigated. Somatic comorbidity was discovered in 999 (98.6%) patients, of which 155 (15.3%) had an isolated pathology, while the vast majority of patients (844 (83.3%) had a concomitant pathology.

The mean CCI value in group 1 was 64%, in group 2−62%, and in group 3−58% with no statistically significant differences between them. It was found that the presence of a concomitant somatic pathology and a patient's age significantly increased postoperative LOS in group 1, while no such effect is observed in groups 2 and 3 (see Table 11).

**Table 11:** CCI effect on postoperative LOS, blood loss, surgical time (rs is Spearman's correlation coefficient, *p* is statistical significance level).

**Parameters**	**Group 1**		**Group 2**		**Group 3**	
**Value**	**Rs**	***p***	**rs**	***P***	**rs**	***p***
LOS	−0.15	0.0006	−0.07	0.22	−0.13	0.35
Surgical time	−0.07	0.11	0.02	0.8	−0.01	0.92
Blood loss	−0.03	0.56	0.04	0.46	0.21	0.14

Correlation analysis of the effect the CCI had on the pain syndrome intensity, functional adaptation, and quality of life in the patients with concomitant pathology at different follow-up times was performed (see Table 12).

**Table 12:** CCI effect on the quality of life (rs is Spearman's correlation coefficient, *p* is statistical significance level).

**Parameters**	**Pre-op**	**0–1 year**	**1–2 years**	**2–3 years**	**3–4 years**
VAS spine	−0.10 (*p =* 0.03)	−0.07 (*p =* 0.19)	−0.10 (*p =* 0.19)	−0.28 (*p =* 0.01)	−0.31 (*p =* 0.006)
VAS leg	−0.07 (*p =* 0.13)	−0.15 (*p =* 0.004)	0.06 (*p =* 0,45)	−0.06 (*p =* 0.62)	−0.21 (*p =* 0.07)
ODI	−0.16 (*p* < 0.001)	−0.13 (*p =* 0.01)	−0.01 (=0,9)	−0.12 (*p =* 0.31)	−0.34 (*p =* 0.002)
SF-36 PH	0.12 (*p =* 0.01)	0.12 (*p =* 0.04)	0.03 (*p =* 0.74)	−0.13 (*p =* 0.4)	0.19 (*p =* 0.2)
SF-36 MH	0.14 (*p =* 0.003)	0,08 (*p =* 0.15)	0.02 (*p =* 0.79)	0.03 (*p =* 0.85)	0.21 (*p =* 0.16)

Table 12 demonstrates that at the preoperative stage the CCI significantly correlates with all the parameters of interest, except for the leg pains primarily associated with nerve root compression in the spinal canal. This correlation indicates the significant effect of somatic comorbidity on quality of life in elderly patients with degenerative lumbar spine pathologies. In addition, the CCI correlated with spinal pain intensity from the third follow-up year onward, as opposed to lower limb pain, which was only associated with the severity of concomitant pathology in the first year after the operation. The statistically significant effect of concomitant pathologies on the physical health indicators (SF-36 PH) and quality of life observed in the first year after the operation was seemingly associated with persistent surgical site pain, motion restrictions, and additional difficulties of the rehabilitation stage in the elderly and geriatric patients with severe somatic pathologies. The severity of the concomitant pathology had no significant effect on the parameters of interest in the second follow-up year due to stabilization of a patient's general condition and lumbar spine condition in particular (which is to be considered as a favorable outcome of surgical treatment). However, the effect of the CCI on the quality of life increased around the fourth follow-up year, which was seemingly associated with the progression of both spinal degenerative changes, including degenerative changes in major lower limb joints, and somatic comorbidity in elderly patients due to aging. All these factors caused loss of functional adaptation and quality-of-life deterioration.

**Figure 1:**
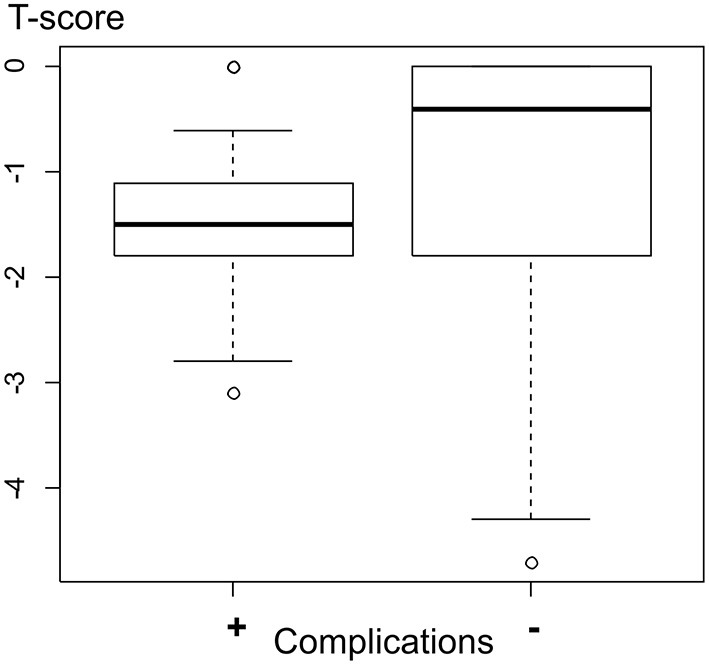
T-score values in patients with complications were −1.41/−1.5 (−1.8; −1.1), and in patients without complications were −0.92/−0.45 (−1.8; 0.0).

The effect of osteoporosis on the quality of life and surgical treatment results was studied in 389 patients who received decompressions with spinal stabilization and corrective operations. Osteoporosis with a T-score below −2.5 was observed in 35 patients (9% of the studied sample), of whom 31 (89%) were females. In all these cases, TPF was combined with polymethylmethacrylate augmentation of the lumbar vertebral bodies via fenestrated screws to increase the resistance of hardware elements to pull-out force. Correlation analysis did not show that osteoporosis had any significant effect on the quality of life in elderly and geriatric patients in the long-term follow-up. However, we identified a significant effect (*p* = 0.045) of the T- score characterizing bone mineral density on the occurrence rates of instrumentation-related complications, such as transpedicular screw malposition, cage migration, and vertebral endplate injuries (see Figure 1).

The T-score values in patients with instrumentation-related complications (*n* = 38) were −1.41/−1.5 (−1.8; −1.1) compared to −0.92/−0.45 (−1.8; 0.0) in patients without said complications (*n* = 351). A statistically significant effect (*p* = 0.045) of T score values characterizing bone mineral density on instrumentation-related complication rate was found. TPF in elderly and geriatric patients was combined with vertebral body augmentation in 9% of cases.

## Discussion

To identify complication predictors as factors of unfavorable outcomes in elderly patients receiving surgical treatment for degenerative lumbar spine pathology, the Dindo-Clavien criteria as a validated and unified assessment tool were applied. Such parameters as the age, gender, BMI, and operation type were analyzed.

To identify complication predictors as factors of unfavorable outcomes in elderly patients receiving surgical treatment for degenerative lumbar spine pathology, the Dindo-Clavien criteria as a validated and unified assessment tool were applied. Such parameters as the age, gender, BMI, and operation type were analyzed.

Any complication per the Dindo-Clavien classification increased the mean LOS regardless of the operation type. Type II and IIIB complications contributed most to increased LOS. Type IIIB complications requiring hospital readmission and reoperation significantly increased the mean LOS and most often developed after TLIF. Meanwhile, type II complications contributed most to increased LOS after indirect decompressions with spinal stabilization and corrective ALIF and LLIF operations.

Our data showed that instrumentation-related complications, such as deep SSI, clinical segmental instability, hardware failure, and Rao grade 3 transpedicular screw malposition, appear to be the most significant complications contributing to increased LOS and, as a result, higher inpatient treatment costs and quality-of-life deterioration in the first 90 days after the initial operation. It is worth noting that decompensated cardiovascular pathology in the early postoperative period significantly increased LOS, which is crucial for elderly patients with comorbidity burden.

A total of 13 patients were readmitted to our center due to complications in 90 days after their initial operations, and 13 more patients underwent redoes within their primary hospital stay. The mean period from the initial operation to hospital readmission was 35 days. The unplanned readmission rate due to complications discovered in the first 90 days in the studied cohort was 1.3%. That can be explained by the use of minimally invasive techniques, which is also confirmed by several authors (33).

The mean LOS was 6.7 days due to the use of MIS techniques in all cases. As a result, we were able to return patients to activity earlier and intensify rehabilitation procedures, thereby reducing the occurrence rate of severe complications in the early postoperative period. The mean intraoperative blood loss was reduced to 178 ml due to available MIS options. Surgical time of over 180 min is considered an important predictor of severe postoperative complications in the lumbar spine in elderly patients (14). The mean surgical time in the studied cohort, including corrective operations for degenerative deformities, was 127 min, which made it possible to minimize the number of complications.

Overall, the occurrence rate of intra- and postoperative complications as per the Dindo-Clavien classification in elderly and geriatric patients with degenerative lumbar spine pathology at 90-day follow-up was 25.1%, which agrees with published literature data (14, 21, 24).

The vast majority of complications in the studied cohort were mild complications (type I, II, and IIIA) accounting for 88.3% of all cases, intraoperative blood loss and dural tear being the most common. The occurrence of complications significantly increased LOS in this category of patients from 6.3/6 (5; 7) (without complications) to 9.3/7 (6; 11) (with complications) (*p* < 0.0001). Increased LOS due to complications is confirmed by the literature data as well (21).

Among several factors affecting complication frequency, the complexity of the surgical procedure was a statistically significant one. Simpler operations have milder complications and lower complication rates (34). MIS TLIF had the highest number of complications compared to decompressions and operations from anterior and lateral approaches since it is the complexity and operation type that determine the surgical time and intraoperative blood loss.

Our data demonstrated the unplanned readmission rate of 1.3% at 90-day follow-up, which is significantly lower than the literature data. The cumulative reoperation rate at 5-year follow-up was 15.2%, and reoperation frequency−12.1%. The analysis of surgical treatment results in 2 320 elderly patients by Saleh et al. showed a readmission rate of 6.39% and complication rate of 16.34%, including fatal outcomes (0.43%) (14). The authors concluded that increased operative times and instrumentation and fusion procedures were strongly associated with an increased risk of developing a complication. However, the authors only analyzed geriatric patients (>80 years) at 30-day follow-up. In addition, the study took into consideration various types of lumbar spine surgical procedures, including primarily open operations, in particular with elongated multilevel fixation hardware. The authors did not use the Dindo-Clavien classification in their complication analysis.

In a multicenter prospective cohort study searched for perioperative complications of spine surgery in 270 patients >80 years of age (35). Overall perioperative complications were observed in 20%, surgical site complications were observed in 8.1%, and minor systemic complications were observed in 14.8% of patients. The reoperation rate was 4.1%. Decreased daily activity, instrumentation surgery, and an operative time >180 min were found to be associated with minor systemic complications. Long fixations resulted in increased morbidity but not mortality.

In Camino Willhuber et al. (21), the reoperation frequency was 11.72%, and the complication rate as per the Dindo-Clavien classification−28.83%. The authors only analyzed elderly patients (with a mean age of 68 years) at 90-day follow-up. Various types of surgical procedures involving all spinal regions were taken into consideration, including primarily open operations not involving MIS techniques. BMI, surgical time, intraoperative blood loss, and operation complexity were identified as complication risk factors in both papers.

Thus, obesity turns out to be among the main predictors of unfavorable surgical outcomes in elderly and geriatric patients with degenerative lumbar spine pathology, even when minimally invasive surgical options are available (36). Increased BMI leads to higher surgical time, which in turn leads to higher intraoperative blood loss and a higher complication rate. The adverse effect of obesity on the occurrence rate of continued degenerative changes in operated segments and adjacent-level pathologies, especially after decompressions with spinal stabilization is not to be overlooked as well. Eventually, this scenario increases the number of unfavorable outcomes and quality-of-life deterioration in both short- and long-term follow-up.

The CCI also has an adverse effect on the quality-of-life indicators, since a high comorbidity burden combined with excess body mass significantly reduces the potential for returning patients to activity in the early postoperative period and favors decompensated cardiovascular pathology and exacerbation of other chronic diseases.

In all elderly patients, avoiding complications related to comorbidities should be the main concern before planning surgery (37). After evaluation of deconditioning, sarcopenia, malnutrition, dementia, and polypharmacy, all measures are implemented in a 6-week presurgical period, including prerehabilitation, nutritional supplementation, medication changes, and preoperative medication for postoperative pain control to minimize delirium. Enhanced recovery after surgery protocols have been shown to be effective at reducing perioperative morbidity and costs, while improving outcomes.

According to meta-analysis some authors recommend that all patients who are >75 years of age be referred to the geriatric medicine clinic for a frailty assessment, because frailty is an important predictor of postoperative complications, increased hospital stays, and mortality (38).

Studying complications in patients of the older age group, we can be concluded that preparation for a planned surgical intervention should begin in advance. It is necessary to take into account all the nuances, including the rejection of certain drugs and diet (39). Despite the fact that some complications, especially those related to the surgical technique, depend only on the experience and actions of the surgeon during the operation, there are a sufficient number of modifiable predictors that can be corrected (40). Understanding this situation allows surgeons to minimize the unfavorable prognosis using an integrated approach to the planned treatment.

Operation complexity also factors in an increased number of unfavorable surgical outcomes in elderly patients with obesity and comorbidity burden, which agrees with the literature data (34). It is worth noting that the occurrence rate of adjacent level pathology increases significantly after rigid segmental fixation, especially in the case of multilevel lesions (36). The more complex and time-consuming an operation is, the higher the complication rate. Therefore, surgical treatment for degenerative lumbar spine pathology in this age group should be decided based on the minimization of surgical aggression (41). Comparable clinical effectiveness is ensured by isolating the prevalent clinical syndrome and selecting a surgical option, including MIS techniques, to reverse the main clinical manifestations of the disease accompanying degenerative pathology.

With the development of advanced image guidance systems, the popularity of minimally invasive procedures has increased. Serious efforts are under way to shorten the learning curve, reduce specific complications make indications more specific, and minimize heterogeneous clinical outcomes. However, minimally invasive techniques in the treatment of LSS are still under development and many guidelines and high-quality studies have been published about the safety and efficacy of these techniques in the past decade (42).

Dagistan et al. examined the effect of minimally invasive decompression surgery on quality of life in 37 elderly patients (between 65 and 86 years old) with spinal stenosis (43). They concluded that decompressive surgeries without instrumentation in elderly patients increased quality of life significantly. The rate of complications was very low. In cases in which complications developed, they could be managed easily. Considering this the surgical intervention itself must be performed using MIS technology, choosing the most effective volume and access in this particular case, which reduces the risk of complications (44, 45).

To improve the outcomes of spine surgery in geriatric patients, Zileli and Dursun also recommend implementation of the following measures: meticulous evaluation of comorbidities, preoperative treatment for some diseases, strict measures to treat osteoporosis, good surgical planning, and use of minimally invasive surgeries as much as possible (38).

This research, however, is subject to several limitations: its retrospective nature, and the limitation to a single center. Also, some patients were lost to the long-term follow-up for various reasons that is a certain limitation which did not allow us to calculate the full percentage of complications on the entire set of patients.

## Conclusions

A registry of postoperative complications is an important tool for health quality assessment and choosing a surgical option that helps to establish measures to reduce such complications. A complication rate depends on several factors such as obesity, comorbidity burden, and operation complexity being the most statistically significant. Using MIS techniques for treating elderly patients reduces the number of severe complications.

## Data Availability Statement

The original contributions presented in the study are included in the article/supplementary materials, further inquiries can be directed to the corresponding author.

## Ethics Statement

The studies involving human participants were reviewed and approved by the Ethics Committee of FSBI Federal Neurosurgical Center Novosibirsk, Russia. The patients/participants provided their written informed consent to participate in this study.

## Author Contributions

VK, AE, and EA contributed to conception and design of the study and organized the database. EA performed the statistical analysis. AE wrote the first draft of the manuscript. All authors contributed to manuscript revision, read, and approved the submitted version.

## Conflict of Interest

The authors declare that the research was conducted in the absence of any commercial or financial relationships that could be construed as a potential conflict of interest.

## Publisher's Note

All claims expressed in this article are solely those of the authors and do not necessarily represent those of their affiliated organizations, or those of the publisher, the editors and the reviewers. Any product that may be evaluated in this article, or claim that may be made by its manufacturer, is not guaranteed or endorsed by the publisher.
